# Proliferin-1 Ameliorates Cardiotoxin-Related Skeletal Muscle Repair in Mice

**DOI:** 10.1155/2021/9202990

**Published:** 2021-11-20

**Authors:** Hiroki Goto, Aiko Inoue, Limei Piao, Lina Hu, Zhe Huang, Xiangkun Meng, Yusuke Suzuki, Hiroyuki Umegaki, Masafumi Kuzuya, Xian Wu Cheng

**Affiliations:** ^1^Department of Community Healthcare & Geriatrics, Nagoya University Graduate School of Medicine, Nagoya, 466-8550 Aichi-ken, Japan; ^2^Institute of Innovation for Future Society, Nagoya University Graduate School of Medicine, Nagoya, 466-8550 Aichi-ken, Japan; ^3^Department of Cardiology and Hypertension, Yanbian University Hospital, Yanjin, 133000 Jilin, China; ^4^Department of Human Cord Applied Cell Therapy, Nagoya University Graduate School of Medicine, Nagoya, 466-8550 Aichi-ken, Japan; ^5^Department of Public Health, Guilin Medical College, Guilin, 541004 Guangxi, China

## Abstract

**Background:**

We recently demonstrated that proliferin-1 (PLF-1) functions as an apoptotic cell-derived growth factor and plays an important role in vascular pathobiology. We therefore investigated its role in muscle regeneration in response to cardiotoxin injury.

**Methods and Results:**

To determine the effects of PLF-1 on muscle regeneration, we used a CTX-induced skeletal muscle injury model in 9-week-old male mice that were administered with the recombinant PLF-1 (rPLF-1) or neutralizing PLF-1 antibody. The injured muscles exhibited increased levels of PLF-1 gene expression in a time-dependent manner. On day 14 after injury, rPLF-1 supplementation ameliorated CTX-induced alterations in muscle fiber size, interstitial fibrosis, muscle regeneration capacity, and muscle performance. On day 3 postinjury, rPLF-1 increased the levels of proteins or genes for p-Akt, p-mTOR, p-GSK3*α*/*β*, p-Erk1/2, p-p38MAPK, interleukin-10, Pax7, MyoD, and Cyclin B1, and it increased the numbers of CD34^+^/integrin-*α*7^+^ muscle stem cells and proliferating cells in the muscles and/or bone marrow of CTX mice. An enzyme-linked immunosorbent assay revealed that rPLF-1 suppressed the levels of plasma tumor necrosis factor-*α* and interleukin-1*β* in CTX mice. PLF-1 blocking accelerated CTX-related muscle damage and dysfunction. In C2C12 myoblasts, rPLF-1 increased the levels of proteins for p-Akt, p-mTOR, p-GSK3*α*/*β*, p-Erk1/2, and p-p38MAPK as well as cellular functions; and these effects were diminished by the depletion of PLF-1 or silencing of its mannose-6-phosphate receptor.

**Conclusions:**

These findings demonstrated that PLF-1 can improve skeletal muscle repair in response to injury, possibly via the modulation of inflammation and proliferation and regeneration, suggesting a novel therapeutic strategy for the management of skeletal muscle diseases.

## 1. Introduction

Aging-related skeletal muscle mass loss and muscle dysfunction can cause reduced quality of life [1]. Accumulating evidence suggests that multiple biological and environmental factors participate in age-associated muscle conditions, including sarcopenia and frailty [2–5]. Various injuries to skeletal muscles can cause muscle weakness and wasting in elderly humans and animals [6–8]. The course of injury-associated skeletal muscle remodeling involves muscle apoptosis and proliferation and the accumulation of extracellular matrix protein, which requires the participation of muscle-resident stem cell (i.e., satellite cell) differentiation and regeneration [9–11]. The bone marrow- (BM-) derived mesenchymal stem cells have also been shown to contribute to skeletal muscle regeneration in animal muscle injury models [12–15]. Many approaches to protect against muscle mass loss and muscle dysfunction have been developed that focus on preventing muscle myofiber loss or stimulating myofiber regeneration (e.g., nutritional, pharmacological, and hormonal approaches), and these approaches are often designed to target muscle cell apoptosis and regeneration mechanisms in aging-associated sarcopenia and frailty [16–18]. Despite important advances, no useful pharmacological therapies that ameliorate or prevent the decline in muscle regeneration and performance in the elderly are currently available for clinical use [3, 19, 20], and this is reflected by ever-increasing social healthcare problems.

Mitogen-regulated proteins, which are also called proliferins (PLFs), are nonclassical members of the family of prolactin/growth hormones that are expressed at high levels by the placenta during pregnancy [21, 22]. The PLF family consists of four homologous members (PLF-related protein and PLF-1, PLF-2, and PLF-3) [23]. In 1995, Nelson et al. identified mannose-6-phosphate receptor (M6pr) as a PLF receptor [24]. Investigations using animal models have led to a number of important findings that have enhanced our understanding of the roles of PLFs and their receptors [25]. For example, it was reported that an extraembryonic protein like PLF-1, which has evolved to support fetal growth, was reactivated in angiogenesis and tumor growth in a cell culture model of fibrosarcoma tumor progression [26]. PLF-1 was also shown to be secreted by Chinese hamster ovary (CHO) cells binding to cation-independent M6prs and targeting lysosomes [27]. By targeting plasminogen to endocytic pathways, M6pr-mediated plasminogen activation can restrict plasmin activity to specific substrates and sites [28]. In endothelial cells, chemotaxis activated by a PLF-1/M6pr axis occurred via a G protein/mitogen-activated protein kinase- (MAPK-) dependent pathway [29]. Jackson and colleagues demonstrated that PLF stimulated placental neovascularization, whereas PLF-related protein inhibited it [30]. In the same study, they reported that PLF and PLF-related protein exhibited the corresponding effects in a rat cornea angiogenesis model [30]. PLF-1 was shown to be required for the nonhypoxic angiogenesis induced by signal transducer and activator of transcription (STAT) 5A signaling pathway activation in endothelial cells [31]. We recently demonstrated an apoptotic cell-derived growth factor (identified as PLF-1) as a communicating mediator between apoptosis and proliferation during vascular remodeling and neointimal formation in mice in response to injuries [32]. However, the role of PLF-1 in muscle repair still remains uncertain.

In the present study, we used the recombinant PLF-1 (rPLF-1) and neutralizing PLF-1 antibody (nPLF-1) and an experimental skeletal injury model to test our hypothesis that PLF-1 modulates skeletal muscle mass and mitigates skeletal muscle wasting in mice in response to injury. We also observed that in C2C12 myoblasts, the depletion of PLF-1 and the silencing of M6pr each decreased targeted growth signaling pathways, providing the first evidence and mechanistic explanation of the involvement of PLF-1 in muscle cell proliferation and the mobilization of bone marrow muscle stem cells (MuSCs) to contribute to muscle repair.

## 2. Materials and Methods

### 2.1. Reagents and Antibodies

The following commercially available antibodies were used. Anti-glyceraldehyde 3-phosphate dehydrogenase (GAPDH; Cat. no. sc-20357) used as a loading control and anti-PLF (sc-271891) were purchased from Santa Cruz Biotechnology (Santa Cruz, CA). p38MAPK, phospho-p38MAPK (p-p38MAPK), mammalian target of rapamycin (mTOR), phospho-mTOR (p-mTOR), extracellular signal-regulated kinase 1/2 (Erk1/2), phospho-Erk1/2 (p-Erk1/2), Akt, phospho-Akt (p-Akt), glycogen synthase kinase 3*α*/*β* (GSK3*α*/*β*), phospho-GSK3*α*/*β* (p-GSK3*α*/*β*), and proliferating cell nuclear antigen (PCNA, PC10) were from Cell Signaling Technology (Beverly, MA). Desmin (Clone 33) was purchased from Dako (Carpinteria, CA). Laminin-5 (BS-7713R) was purchased from Bioss (Woburn, MA). Zenon mouse and rabbit IgG labeling kits were purchased from Molecular Probes (Eugene, OR). The fluorescein isothiocyanate- (FITC-) labeled CD34 antibody was purchased from eBioscience (San Diego, CA). The phycoerythrin-labeled integrin-*α*7 antibody was purchased from Medical & Biological Laboratories Co. (Nagoya, Japan). The Ki67 antibody was from Lab Vision/NeoMarkers (Fremont, CA). Mouse control IgG and biotinylated mouse nPLF-1 were from R&D Systems (Minneapolis, MN).

The following commercially available reagents were used. Horse serum was purchased from GIBCO Life Technologies (Auckland, New Zealand). The RNeasy Fibrous Tissue Mini Kit was from Qiagen (Hilden, Germany). The Universal polymerase chain reaction (PCR) Master Mix and Core Kit were from Applied Biosystems (Foster City, CA). Cardiotoxin (CTX) was from Sigma-Aldrich (Cat. Naja pallida, L8102, Latoxan and Naja mossambica C9759). Dulbecco's modified Eagle's medium (DMEM) was from GIBCO Life Technologies (Grand Island, NY). The optimal cutting temperature (OCT) compound was from Sakura Finetechnical (Tokyo). The Amersham ECL Prime Western Blotting Detection kit was from GE Healthcare (Freiburg, Germany). The FreeStyle™ MAX CHO Expression System (Cat. K900020: including the CHO cells, FreeStyle™ MAX reagent, and OptiPRO™ SFM), Lipofectamine RNAiMAX reagent, and Lipofectamine LTX & Plus reagents were from Invitrogen (Carlsbad, CA). The CellTiter 96 AQ Assay kit was from Promega (Madison, WI). The short interfering RNA against M6pr (siM6pr; Mm_m6pr_3685-a, Mm_m6pr_3685-as), the nontargeting control siRNA (Mission_SIC-001_s and Mission_SIC-001_as), and the mouse interleukin-1*β* (IL-1*β*) enzyme-linked immunosorbent assay (ELISA) kit were from Sigma-Aldrich (St. Louis, MO). The silamin A/C (D0010500105) was from Dharmacon (Brébières, France). The mouse tumor necrosis factor-alpha (TNF-*α*) ELISA kit was from R&D Systems (Minneapolis, MN). The ABC substrate kit (SK-4400) was from Vector Laboratories (Burlingame, CA). C2C12 mouse myoblasts were purchased from the American Type Culture Collection (Manassas, VA).

### 2.2. Mice

Nine-week-old male mice (C57BL/6 background) were purchased from SLC (Hamamatsu, Japan). They were fed a standard diet and housed three per cage under standard conditions (22° ± 2°C, 50% ± 5% humidity) with a 12 hr light-dark cycle at the Animal Research Center of the Nagoya University Graduate School of Medicine. All animal protocols were approved by the Institutional Animal Care Committee of Nagoya University (Protocol No. 30122) and were conducted according to the *Guide for the Care and Use of Laboratory Animals* published by the U.S. National Institutes of Health.

### 2.3. Animal Experiments and Tissue Collections

A CTX-induced muscle injury model was created as described previously [8]. In brief, the left leg hair of a 10-week-old male mouse was shaved, and the mouse was injected into the left gastrocnemius muscle with CTX solution (20 *μ*M/100 microliter) and then subjected to muscle biological (PLF-1 expression), morphological (muscle injury degree), and functional (grip strength and endurance capacity) assessments at the indicated timepoints. For evaluation of the efficacy of rPLF-1, mice were injected subcutaneously with the vehicle (saline) or rPLF-1 (150 *μ*g/kg/d) on days −1, 1, 3, 5, and 7 after the injury [32]. In a separate neutralizing antibody study, mice were injected subcutaneously with either the mouse control IgG or the biotinylated mouse monoclonal antibody (mAb) against nPLF-1 (450 *μ*g/kg/d) as indicated.

Following a muscle performance test, the mice were anesthetized with an intraperitoneal injection of pentobarbital sodium (50 mg/kg), and the blood samples and the tissues were isolated at the indicated timepoints. The gastrocnemius muscle was sampled and kept in RNAlater solution for the gene assay or in liquid nitrogen for the protein assay. After being immersed in a fixative at 4°C, the muscle tissues were embedded in the OCT compound and stored at −20°C for histological analysis.

### 2.4. Grip Strength Evaluation

The grip strength of the mice was evaluated as described previously [3]; in brief, the mouse limbs were placed on the limb grip of a small-animal grip strength meter (Columbus, Largo, FL), and then the mouse's tail was gently pulled in the opposite direction. We calculated the maximum value of the grip force before it released its grip. The grip strength was calculated >5 times and averaged as the expression of grip strength for each mouse on days 0, 3, and 14. The mice that underwent endurance and grip strength evaluations were excluded from the biological and histological assays in order to exclude the function testing's influence.

### 2.5. Skeletal Muscle Endurance Capacity and Grip Strength

For the skeletal muscle performance assay, we used a motorized rodent treadmill (S-Con Mini-Z; Tokyo Engineering, Tokyo) to evaluate the endurance ability of the mice as described previously [3]: in brief, mice on days 3 and 14 after the CTX injection were put on the treadmill, and the warm-up was started at 6 m/min with the treadmill's tilt angle at 0°. Following the 5 min warm-up, the tilt angle of the treadmill was upped to 10° and the speed was gradually increased by 2 m/min every 2 min, and the speed was kept at the maximum speed of 20 m/min. The counting of the distance and running workload were stopped when the mouse rested for >10 sec. The running distance was calculated as the product of the running time and the running speed. The workload in the vertical direction was calculated with consideration for the mouse body weight (workload in the vertical direction = body weight × gravitational acceleration × mileage in the vertical direction). The endurance capacity was expressed as the ratio of the calculated values to the data obtained on day 0 before the CTX injury.

### 2.6. Western Blot Assay

For the western blot assay, after the extraction of total protein from the tissues and the lysates with a RIPA lysis buffer, equal amounts of protein (40 *μ*g/line) were transferred to polyvinylidene difluoride membranes and immunoreacted with the following targeted primary antibodies: IL-10, total Akt, p-Akt, total mTOR, p-mTOR, total p38MAPK, p-p38MAPK, p-GSK3*α*/*β*, total GSK3*α*/*β*, total Erk1/2, p-Erk1/2, and GAPDH (1 : 1,000 for each antibody) [33]. The determination of targeted proteins was performed using an Amersham ECL Prime Western Blotting Detection kit. Quantifications of targeted protein amounts from western blots were normalized by loading internal GAPDH as the control.

### 2.7. Immunohistochemistry and Morphometry Assays

Serial cross-cryosections (4 *μ*m) were obtained at a ratio of 3–4 sections every 40 *μ*m at the damaged regions of the gastrocnemius muscle. On day 14 postinjury, the sections were immunostained and incubated with the mouse monoclonal antibody against PCNA (1 : 50), and the cell proliferation was visualized with an ABC substrate kit.

For the evaluation of the gastrocnemius muscle myofiber size, the sections at 14 days postinjury were stained with H&E. For the muscle fibrosis assay, the sections from the gastrocnemius muscles on day 14 postinjury underwent Masson's trichrome staining. We took 6–7 images of single sections using a ×20 objective, and we calculated the numbers of PCNA^+^ cells for the quantification of positve staining cells. For the quantifications of fibrosis and muscle myofibers, we took 6–7 images at 9 × 10^4^ *μ*m^2^ for single sections using a ×20 objective, and we determined the volume of interstitial fibrosis and the average size of the muscle fibers with the central nucleus in each field using a fluorescence microscope (BZ9000; Keyence, Osaka, Japan). For negative controls, the first antibodies were replaced with Zenon-labeled rabbit or mouse IgG or nonimmune immunoglobulin G.

### 2.8. Gene Expression Assay

RNA was isolated from the lysates or tissue with an RNeasy Fibrous Tissue Mini Kit. An RNA PCR Core Kit was applied for the mRNA reverse transcription to cDNA. A quantitative real-time PCR was done using the Universal PCR Master Mix with an ABI 7300 PCR system (Applied Biosystems). All analyses were performed in triplicate. The sequences of the primers used for GAPDH, Pax7, MyoD, and Cyclin B1 genes are provided elsewhere [34]. The transcription of target genes was normalized by the GAPDH gene.

### 2.9. Immunofluorescence Assay

For double immunofluorescence, the gastrocnemius muscle was cryosectioned at 4 *μ*m thickness on the indicated days. The sections were treated with anti-desmin and anti-laminin-5 (1 : 100 for each), and then the sections were incubated with the Zenon rabbit and mouse IgG labeling kits (1 : 200). The positively stained sections were observed with a fluorescence microscope (BZ-X700; Keyence). We evaluated the average intensity of desmin for 6–8 fibers in 1 section by using the ImageJ software program (U.S. NIH).

### 2.10. The ELISA and Biological Analysis

Blood was obtained directly from the left ventricles of mice for the ELISA and biological analyses. The plasma IL-1*β* and TNF-*α* levels were evaluated using an ELISA kit according to the manufacturer's instructions.

### 2.11. BMSC Mobilization Assay

At day 14 after CTX injection, BM and peripheral blood (PB) samples were obtained from the two experimental groups, and erythrocytes were lysed with ammonium chloride and separated into pellets. The cells were washed with PBS and sorted by flow cytometry using fluorescein isothiocyanate- (FITC-) labeled CD34 and phycoerythrin-labeled integrin-*α*7 as described [35].

### 2.12. rPLF-1 Production and Purification

For the production of mouse rPLF-1, we used a FreeStyle™ MAX CHO Expression System to generate rPLF-1 as described [32]. Briefly, CHO cells were incubated in the FreeStyle CHO Expression Medium containing 0.5x Pen-Strep and 8 mM of L-glutamine in a 37°C incubator containing a humidified atmosphere of 8% CO_2_ in air with shaking at 120 rpm/min. CHO cells at 1–1.5 × 10^6^ cells/mL were diluted in the FreeStyle CHO Expression Medium at 1 × 10^6^/mL and then subjected to the transfection procedure. Next, 37.5 *μ*L of the FreeStyle™ MAX reagent was diluted with 0.6 mL of OptiPRO™ SFM (serum-free medium), and 37.5 *μ*g of the pcDNA3.1-PLF-Flag plasmid was diluted in 0.6 mL of OptiPRO™ SFM.

The diluted FreeStyle MAX Transfection Reagent was then mixed with the diluted DNA solution and treated for 10 min at 37°C. The DNA-FreeStyle MAX Reagent complex and CHO cells were mixed in a flask (total cells: 1 × 10^7^/30 mL) and allowed to culture continuously while monitoring the mouse PLF-1 protein expression levels. On day 3 posttransfection, the media were isolated by centrifugation (1,000 rpm) for 5 min and stored at −80°C. The rPLF-1 purification and lyophilization were performed by Invitrogen (Life Technologies, Carlsbad, CA).

### 2.13. Cell Culture

C2C12 mouse myoblasts were grown in Dulbecco's modified Eagle's medium (DMEM; GIBCO Life Technologies, Grand Island, NY, USA) containing 10% (vol/vol) fetal bovine serum (FBS) and antibiotics at 37°C with 5% CO_2_. The C2C12 myoblasts were grown on 60 mm dishes until 50% confluence and were subjected to M6pr gene silencing and PLF-1 gene overexpression experiments as below.

### 2.14. Target Gene Silencing and Overexpression Experiments

For silencing of the M6pr gene, C2C12 myoblasts were cultured on 60 mm dishes until 50% confluent. The siM6pr (Mm_m6pr_3685-a, Mm_m6pr_3685-as) or control siRNA (Mission_SIC-001_s and Mission_SIC-001_as) mixed with the antibiotic-free DMEM-2 medium containing the Lipofectamine RNAiMAX reagent, respectively, was added to each cultured well to reach a final siM6pr concentration of 100 pM, and the cells were then continuously cultured for 48 hr for the targeted gene assay as described [32]. Transfected cells were also used for cell migration and proliferation assays. Silamin A/C was used as a positive control.

For the overexpression experiments, the C2C12 myoblasts were cultured on 10 cm dishes at a density of 4 × 10^6^ cells/dish and cultured in MEM supplemented with antibiotic-free 10% FBS overnight. The cells were then transfected with the PLF-1 plasmid (pl-PLF-1; pcDNA3.1-Flag, pcDNA3.1-PLF-Flag, pAAV-IRES-hrGFP, and pAAV-PLF-IRES-hrGFP plasmids) using the Lipofectamine 2000® transfection reagent and cultured for 48 hr [32]. A vehicle (culture medium, N-C) and a mimic control (Lipofectamine transfection reagent only, Lip) were added. The transfected cells were applied to the assays of gene expression, proliferation, and western blotting.

### 2.15. Assay of Cell Proliferation, Migration, and Invasion

Cell proliferation was investigated with a CellTiter 96 AQ Assay kit as described previously [36]. In brief, the C2C12 myoblasts were transfected with siM6pr for 48 hr. To each well of a collagen-coated 96-well plate, 5 × 10^3^ cells, 100 *μ*L of 0.3% BSA/DMEM, and EBM-2 containing rPLF-1 (50 ng/mL) were added into each well, and then the plate was incubated for 24 hr. Then, 20 *μ*L of a mixture of the phenazine methosulfate and tetrazolium compound was added, and the absorbance was measured at 492 nm. Proliferation experiments were done four separate times for each group in triplicate.

For migration assay, 100 *μ*g/mL type collagen-coated and invasion assays were done on Transwell 24-well tissue culture plates as described previously [32]. The C2C12 myoblasts that migrated (100 *μ*g/mL type collagen-coated Transwell membrane) and invaded (1 mg/mL type collagen gel Transwell membrane) to the outer side of the membrane were stained and calculated in 5–7 randomly chosen fields of the duplicated chambers at a magnification of ×200 for each sample.

### 2.16. Statistical Analysis

Data are presented as mean ± SE. Student's *t*-test for comparisons between groups and one-way analysis of variance (ANOVA) for comparisons of three or more groups followed by Tukey's post hoc tests were used for statistical analyses. The grip strength and workload change data were subjected to two-way repeated-measures ANOVA and Bonferroni's post hoc tests. The myofiber size, desmin intensity, and number of PCNA^+^ proliferative cells were evaluated by two observers in a blind manner, and the values they obtained were averaged. SPSS software ver. 17.0 (SPSS, Chicago, IL) was used. Probability (*p*) values < 0.05 were considered significant.

## 3. Results

### 3.1. Changes in PLF-1 Expression, Myofiber Size, Fibrosis, and Muscle Performance in response to CTX Injection

**Figure 1:**
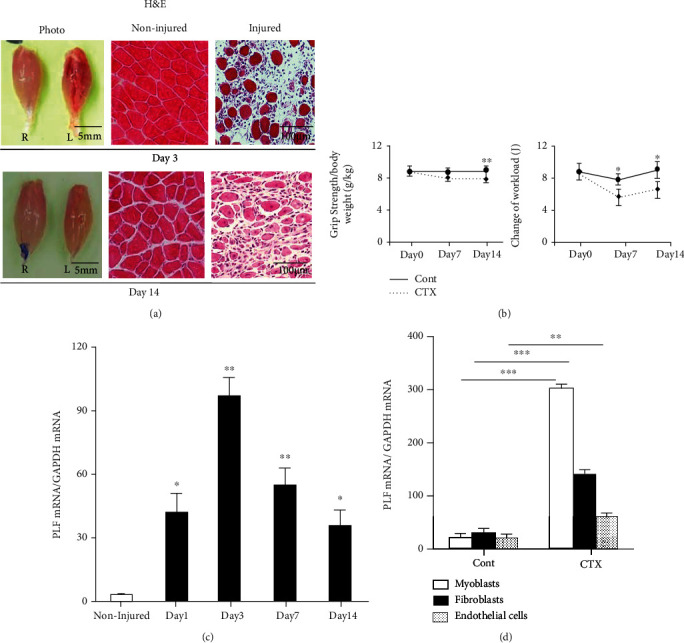
Expressions of PLF-1 in the gastrocnemius muscles at the indicated timepoints after cardiotoxin (CTX) injection. (a) Photos of a gastrocnemius mass and representative microscopy images of H&E staining of the noninjured and injured muscles of mice on days 3 and 14 postinjury. Grip strength was calculated in both groups. (b) The changes of workload in the vertical direction were evaluated in both experimental groups as described in Materials and Methods. (c) Quantitative real-time PCR data show the levels of PLF-1 on days 0, 1, 3, 7, and 14 postinjury. (d) Real-time PCR data showed the PLF-1 gene expression in C2C12 myoblasts, fibroblasts, and endothelial cells inducted by CTX at 10 *μ*M. Results are mean ± SE (*n* = 6–7). ^∗^*p* < 0.05, ^∗∗^*p* < 0.01, and ^∗∗∗^*p* < 0.001 vs. the corresponding day 0 by one-way ANOVA followed by Tukey's post hoc tests.

Figures 1(a) and 1(b) show severely damaged gastrocnemius muscles (e.g., muscle fiber loss, hemorrhage, and edema) and the decline in the grip strength and workload in CTX-injected mice. As a first step to evaluate the PLF-1 expression in response to CTX injury, we extracted total RNA from the noninjured and injured muscles at the indicated timepoints after CTX injection and performed a quantitative real-time PCR assay to determine the PLF-1 mRNA levels. We observed only a low level of PLF-1 gene expression in the noninjured muscle tissues (Figure 1(c)). In contrast, the PLF-1 gene levels were markedly elevated in the CTX-injected muscles throughout the follow-up period and reached a peak on day 3 postinjury (Figure 1(c)). In the in vitro experiments, cardiotoxin increased PLF-1 mRNA expression in the mouse C2C12 myoblasts, fibroblasts, and endothelial cells, and the highest expression of PLF-1 mRNA was observed in CTX-treated C2C12 myoblasts (Figure 1(d)), suggesting that apoptotic skeletal muscles may be one of the major cell sources of PLF-1 production in the injured muscle tissues under our experimental conditions.

### 3.2. Administration of rPLF-1 Prevented Muscle Damage and Dysfunction in response to CTX

**Figure 2:**
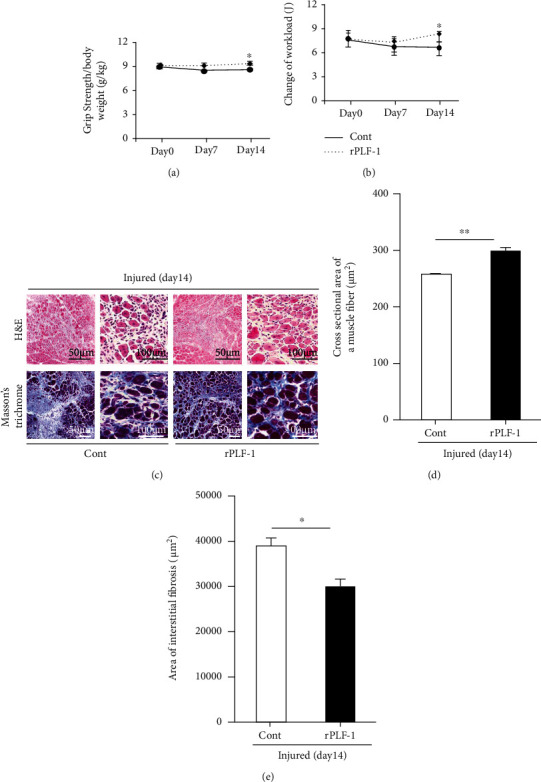
Recombinant proliferin-1 (rPLF-1) ameliorated skeletal muscle dysfunction and remodeling on day 14 after cardiotoxin (CTX) injection. (a) Grip strength was calculated in both groups. (b) The changes of workload in the vertical direction were evaluated in both experimental groups as described in Materials and Methods. (c-e) Quantitative data showing the cross-sectional area of the myofiber size and interstitial fibrosis (9 × 10^4^ *μ*m^2^). Results are mean ± SE (*n* = 6–7). ^∗^*p* < 0.05 and ^∗∗^*p* < 0.01 by two-way repeated-measures ANOVA and Tukey's post hoc tests or Student's *t*-test.

As a second step to examine whether the administration of rPLF-1 protects against muscle mass loss and fibrosis, we developed a model of skeletal muscle CTX injury using mice treated with the vehicle (saline) or rPLF-1 (150 *μ*g/kg/d) at the indicated timepoints to monitor muscle functional and morphological alterations in the muscle tissues. The quantitative data of muscle performance on day 14 postinjury revealed that rPLF-1 ameliorated the impaired workload and grip strength in CTX-injured mice (Figures 2(a) and 2(b)). The quantitative morphological data demonstrated that the rPLF-1 mice had better preserved myofiber sizes (298 ± 6.4 vs. 255 ± 3.4 *μ*m^2^, *p* < 0.01) and lower levels of interstitial fibrosis (30594 ± 1261 vs. 38536 ± 2302 *μ*m^2^, *p* < 0.05) compared to the control mice, respectively (Figures 2(c)–2(e)).

### 3.3. rPLF-1 Ameliorated Muscle Inflammation and Muscle Regeneration via Bone Marrow-Derived MuSC Production and Mobilization in response to CTX

**Figure 3:**
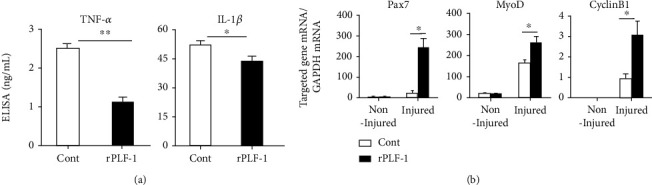
rPLF-1 ameliorated inflammation in response to CTX injury. (a) ELISA data show the levels of plasma TNF-*α* and IL-1*β* in both experimental groups on day 3 after CTX injection. (b) Quantitative real-time data show the levels of Pax7, MyoD, and Cyclin B1 mRNA expressions in the gastrocnemius muscles of both groups of mice. Results are mean ± SE (*n* = 6–7). ^∗^*p* < 0.05 and ^∗∗^*p* < 0.01 by Student's *t*-test or one-way ANOVA followed by Tukey's post hoc tests.

**Figure 4:**
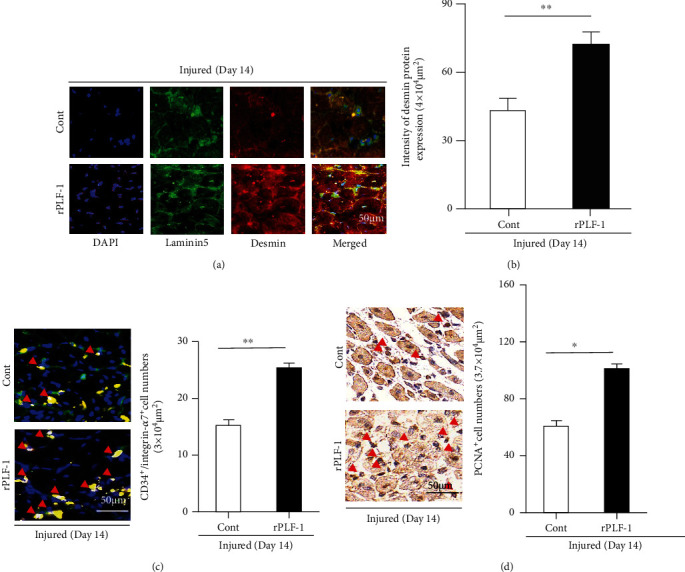
rPLF-1 alleviated the expressions of desmin and laminin proteins in the gastrocnemius muscles at the indicated days after CTX injection. (a, b) Double immunofluorescence was performed with the mouse mAb against desmin (*red*) and the rabbit polyclonal antibody (pAb) against laminin-5 (*green*). Representative images and quantitative data of the desmin protein expression in the gastrocnemius muscle of both experimental groups. (c) Double immunofluorescence was performed with goat pAb against integrin-*α*7 (*red*) and rabbit mAb against CD34 (*green*). Representative images and quantitative data show the numbers of CD34^+^/integrin-*α*7^+^ muscle stem cells (MuSCs). (d) Immunostaining was performed with mouse mAb against mouse monoclonal proliferating cell nuclear antigen (PCNA). Representative images and quantitative data show PCNA-positive cells. *Red arrowheads*: CD34^+^/integrin-*α*7^+^ cells or PCNA^+^ cells. Data are mean ± SE (*n* = 6–7). ^∗^*p* < 0.05 and ^∗∗^*p* < 0.01 vs. controls by Student's *t*-test.

It has been established that inflammatory cytokines/chemokines play a pivotal role in all stages of muscle wound healing after injury [8]. In our present experiments, because muscle damage and mass loss seemed to be closely associated with increased inflammatory cytokines, we extended our examination of the injury healing process to the inflammatory cytokine production on day 3 postinjury. The results indicated that rPLF-1 ameliorated the plasma TNF-*α* and IL-1*β* levels (TNF-*α*: 1.1 ± 0.1 vs. 2.5 ± 0.1 ng/mL, *p* < 0.01; IL-1*β*: 43.7 ± 2.7 vs. 52.7 ± 2.4 ng/mL, *p* < 0.05 for each) (Figure 3(a)), suggesting that rPLF-1 may have an anti-inflammatory property. The real-time PCR using muscles from both experimental groups showed that the injured muscles of the rPLF-1-treated mice had increased levels of Pax7, MyoD, and Cyclin B1 genes (Figure 3(b)). To further visualize the regeneration process, we performed immunostaining for desmin, an intermediate filament protein highly expressed in immature muscle fibers during fetal life and regeneration [7]. Here, we applied double immunofluorescence using laminin-5 and desmin antibodies to visualize the regeneration process. As seen in Figures 4(a) and 4(b), the injured gastrocnemius muscle on day 14 after CTX injury showed low desmin expression (intracellularly scattered staining signals). In contrast, desmin protein expression (intracellularly diffused strong staining signals) was dramatically increased in the CTX-injured muscle on day 14 after rPLF-1 treatment (71.9 ± 5.9 vs. 43.6 ± 3.5, *p* < 0.01) compared to the control mice, which indicates that rPLF-1 supplementation prevented the muscle fiber damage and restored healing in response to the CTX injection.

**Figure 5:**
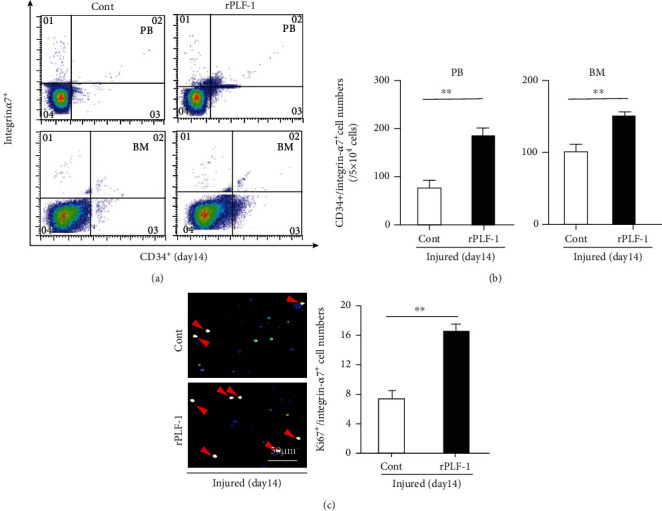
rPLF-1 stimulated bone marrow (BM) MuSC production and mobilization in response to CTX injury. (a, b) Representative dot plots and quantitative data for the numbers of CD34^+^/integrin-*α*7^+^ MuSCs in BM and peripheral blood (PB). (c) After the isolation of BM-derived integrin-*α*7^+^ stem cells with magnetic beads, the cells were cultured on cover glasses for 24 hr and then subjected to double immunofluorescence with goat pAb against integrin-*α*7 (*red*) and mouse mAb against Ki67 (*green*). Representative double fluorescence images and quantitative data show the numbers of proliferating cells (×200 magnification). Results are mean ± SE (*n* = 7–8). ^∗∗^*p* < 0.01 vs. corresponding control groups by Student's *t*-test.

In agreement with these findings, the numbers of CD34^+^/integrin-*α*7^+^ MuSCs were much higher (25 ± 1.5 vs. 15 ± 0.8, *p* < 0.01) in the gastrocnemius muscles of the rPLF-1 mice compared to the control mice (Figure 4(c)). The quantitative data of the flow cytometry analysis demonstrated that the rPLF-1 treatment resulted in elevated numbers of CD34^+^/integrin-*α*7^+^ MuSCs in the bone marrow and peripheral blood (Figures 5(a) and 5(b)). Double immunofluorescence showed that the numbers of Ki67^+^/integrin-*α*7^+^ cells were significantly higher (17 ± 0.7 vs. 7.5 ± 0.3, *p* < 0.01) in the bone marrow of the rPLF-1 mice compared to the control mice (Figure 5(c)). These results suggested that rPLF-1 can increase MuSC production and mobilization in this mouse model, leading to muscle regeneration under our experimental conditions.

### 3.4. rPLF-1 Promoted Cell Proliferation via the Activation of Growth Signaling in CTX-Injured Muscles

**Figure 6:**
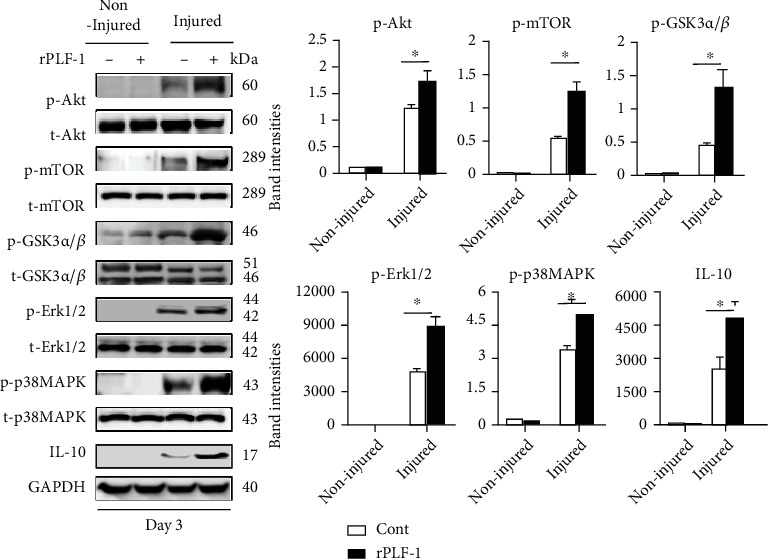
rPLF-1 increased the levels of proliferation-related signal proteins in the gastrocnemius muscle in response to CTX injury. Representative immunoblots and combined quantitative data show increased levels of p-Akt, p-mTOR, p-GSK3*α*/*β*, p-Erk1/2, p-p38MAKP, and IL-10 in the muscles of rPLF-1 mice. Results are mean ± SE (*n* = 3). ^∗^*p* < 0.05 and ^∗∗^*p* < 0.01 vs. corresponding control groups by one-way ANOVA followed by Tukey's post hoc tests.

PLF-1 growth signaling has been shown to participate in angiogenesis in tumor growth [26]. In our present experiments, quantitative immunostaining revealed that rPLF-1 treatment increased the number of PCNA^+^ cells in the injured gastrocnemius muscles (Figure 4(d)). Immunoblot analysis using equal amounts of protein from each sample showed marked increases by the gel density analyses of the growth signaling proteins (p-Akt, p-mTOR, p-GSK3*α*/*β*, p-Erk1/2, p-p38MAPK, and IL-10) in the rPLF-1-treated mice (Figure 6), suggesting that PLF-1-mediated growth signaling might contribute to the prevention of muscle mass loss in response to CTX injury.

### 3.5. PLF-1 Depletion Accelerated the CTX-Induced Muscle Damage and Dysfunction

**Figure 7:**
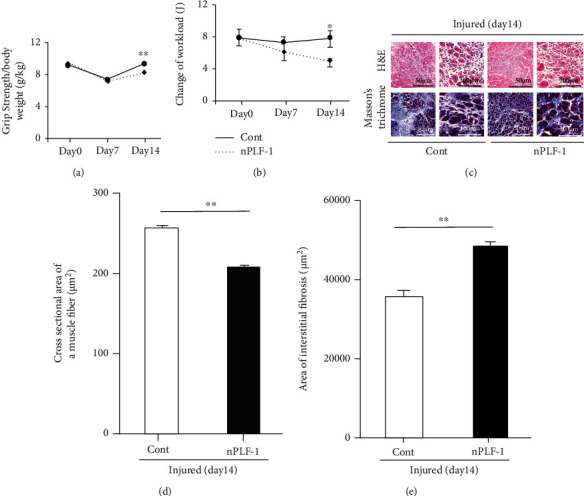
PLF-1 blocking accelerated skeletal muscle dysfunction and remodeling on day 14 after CTX injection. (a) Grip strength was calculated in both groups. (b) The changes of workload in the vertical direction were evaluated in both groups as described in Materials and Methods. (c-e) Quantitative data showing the cross-sectional area of muscle fiber size and interstitial fibrosis (9 × 10^4^ *μ*m^2^). Results are mean ± SE (*n* = 6–7). ^∗^*p* < 0.05 and ^∗∗^*p* < 0.01 by two-way repeated-measures ANOVA and Tukey's post hoc tests or Student's *t*-test.

To further investigate the role of PLF-1 in the regulation of muscle mass, we conducted a PLF-1 blocking experiment using a neutralizing antibody against PLF-1 in the same injury model. The results showed that PLF-1 depletion markedly accelerated the impaired grip strength and endurance capacity of the mice on day 14 postinjury (Figures 7(a) and 7(b)). The myofiber sizes were significantly smaller (206 ± 2.5 vs. 254 ± 3.6 *μ*m^2^, *p* < 0.01), and the amount of interstitial fibrosis was significantly higher (47884 ± 1032 vs. 35290 ± 964 *μ*m^2^, *p* < 0.01) in the gastrocnemius muscles of the nPLF-1-treated mice compared to the control mice (Figures 7(c)–7(e)).

### 3.6. PLF-1 Depletion Accelerated Muscle Inflammation and Delayed the Muscle Regeneration in response to CTX

**Figure 8:**
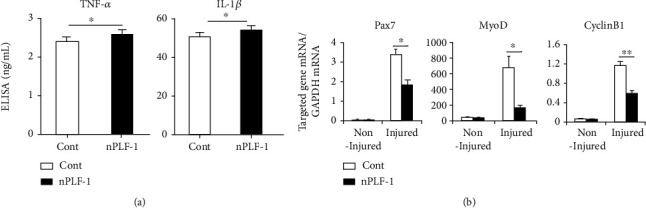
PLF-1 blocking accelerated the inflammation in response to CTX injury. (a) ELISA data show the levels of plasma TNF-*α* and IL-1*β* in both experimental groups on day 3 after CTX injection. (b) Quantitative real-time data show the levels of Pax7, MyoD, and Cyclin B1 mRNA expressions in the gastrocnemius muscles of both groups of mice. Results are mean ± SE (*n* = 6–8). ^∗^*p* < 0.05 and ^∗∗^*p* < 0.01 by Student's *t*-test or one-way ANOVA followed by Tukey's post hoc tests.

**Figure 9:**
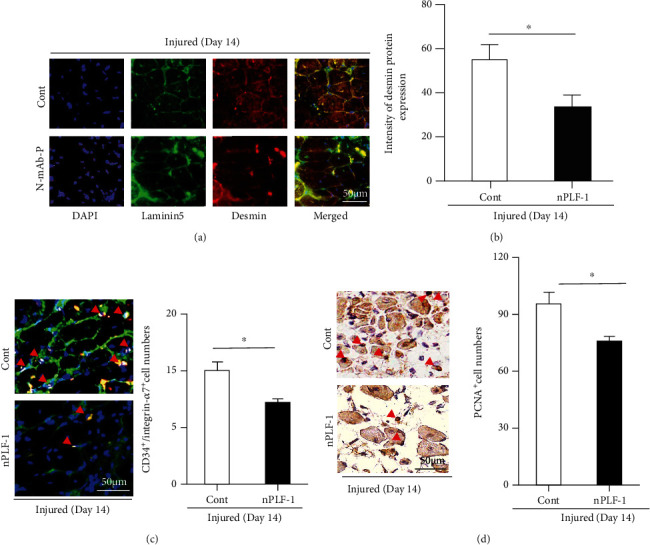
PLF-1 blocking accelerated the impaired desmin and laminin expressions in the gastrocnemius muscles postinjury. (a, b) Double immunofluorescence was performed with the mouse monoclonal antibody (mAb) against desmin (*red*) and the rabbit polyclonal antibody (pAb) against laminin-5 (*green*). Representative images and quantitative data show the contents of desmin proteins in the gastrocnemius of both experimental groups. (c) Double immunofluorescence was performed with goat pAb against integrin-*α*7 (*red*) and rabbit mAb against CD34 (*green*). Representative images and quantitative data show the numbers of CD34^+^/integrin-*α*7^+^ muscle stem cells (MuSCs) in the gastrocnemius muscles of both experimental groups. (d) Immunostaining was performed with mouse mAb against proliferating cell nuclear antigen (PCNA). Representative images and quantitative data show PCNA-positive cells in the gastrocnemius muscles of both groups. *Red arrowheads*: CD34^+^/integrin-*α*7^+^ cells or PCNA^+^. Data are mean ± SE (*n* = 7–8). ^∗^*p* < 0.05 and ^∗∗^*p* < 0.01 vs. controls by Student's *t*-test.

**Figure 10:**
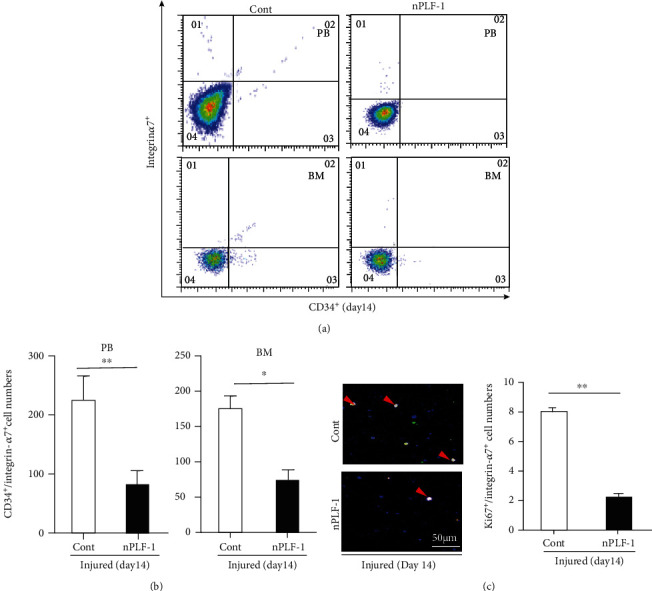
PLF-1 depletion reduced the numbers of MuSCs in bone marrow (BM) and peripheral blood (PB) in response to CTX injury. (a, b) Representative dot plots and quantitative data for the numbers of CD34^+^/integrin-*α*7^+^ MuSCs in BM and PB of both experimental groups. (c) After the isolation of BM-derived integrin-*α*7^+^ stem cells with magnetic beads, the cells were cultured on cover glasses for 24 hr and then subjected to double immunofluorescence with goat pAb against integrin-*α*7 (*red*) and mouse mAb against Ki67 (*green*). Representative double images and quantitative data show the numbers of proliferating cells (×200 magnification). Results are mean ± SE (*n* = 6–8). ^∗∗^*p* < 0.01 vs. corresponding control groups by Student's *t*-test.

As shown in Figure 8(a), PLF-1 blocking significantly elevated the plasma TNF-*α* and IL-1*β* levels (TNF-*α*: 2.66 ± 0.02 vs. 2.45 ± 0.09 ng/mL; IL-1*β*: 53.5 ± 1.4 vs. 47.7 ± 1.1 ng/mL; *p* < 0.05 for each) in CTX-injured mice. As anticipated, nPLF-1 also inhibited the levels of Pax7, MyoD, and Cyclin B1 gene expression in the injured gastrocnemius muscles (Figure 8(b)). Interestingly, we observed that nPLF-1 significantly suppressed the desmin protein expression (34.0 ± 1.4 vs. 56.1 ± 4.7, *p* < 0.05) and the numbers of CD34^+^/integrin-*α*7^+^ MuSCs (11.5 ± 0.5 vs. 15.0 ± 0.8, *p* < 0.05) in CTX-injured gastrocnemius muscles (Figures 9(a)–9(c)). The quantitative flow cytometry analysis yielded similar conclusions. The nPLF-1-treated mice exhibited significantly lower levels of CD34^+^/integrin-*α*7^+^ MuSCs in peripheral blood (69.5 ± 17.8 vs. 235.5 ± 36.1, *p* < 0.01) and bone marrow (80.3 ± 15.7 vs. 177.5 ± 22.1, *p* < 0.05) compared to the control mice (Figures 10(a) and 10(b)). The double immunofluorescence analysis also revealed that PLF-1 blocking resulted in significantly decreased numbers of Ki67^+^/integrin-*α*7^+^ cells (2.3 ± 0.3 vs. 8.5 ± 0.3, *p* < 0.01) in the bone marrow of the CTX mice (Figure 10(c)).

### 3.7. nPLF-1 Inhibited Proliferation via the Inactivation of Growth Signaling in CTX-Injured Muscles

**Figure 11:**
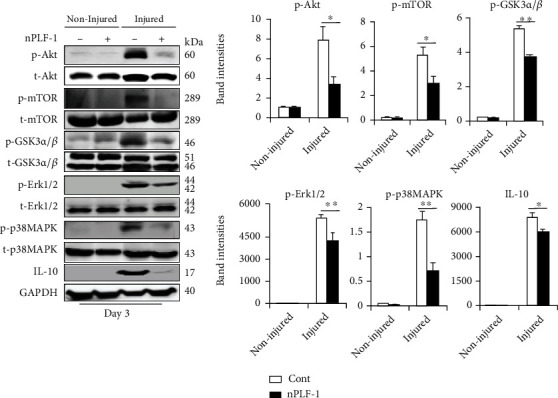
PLF-1 depletion accelerated the impaired proliferation signaling activation in the gastrocnemius muscle in response to CTX injury. Representative immunoblots and combined quantitative data show decreased levels of p-Akt, p-mTOR, p-GSK3*α*/*β*, p-Erk1/2, p-p38MAPK, and IL-10 in the muscles of nPLF-1-treated mice. Results are mean ± SE (*n* = 3). ^∗^*p* < 0.05 and ^∗∗^*p* < 0.01 vs. corresponding control groups by one-way ANOVA followed by Tukey's post hoc tests.

As shown in Figure 9(d), PLF-1 blocking decreased the numbers of PCNA^+^ cells in the injured gastrocnemius muscles (81.3 ± 3.5 vs. 98.8 ± 4.1, *p* < 0.05). Consistent with this finding, the quantitative data from the western blot analysis showed that PLF-1 blocking dramatically reduced the levels of the targeted growth signaling molecules (p-Akt, p-mTOR, p-GSK3*α*/*β*, p-Erk1/2, p-p38MAPK, and IL-10) (Figure 11), suggesting that PLF-1 blocking exerted a harmful effect on cell proliferation in the skeletal muscle in response to injury.

### 3.8. A PLF-1/M6pr Axis Was Required in the Growth Signaling Activation in C2C12 Cells

**Figure 12:**
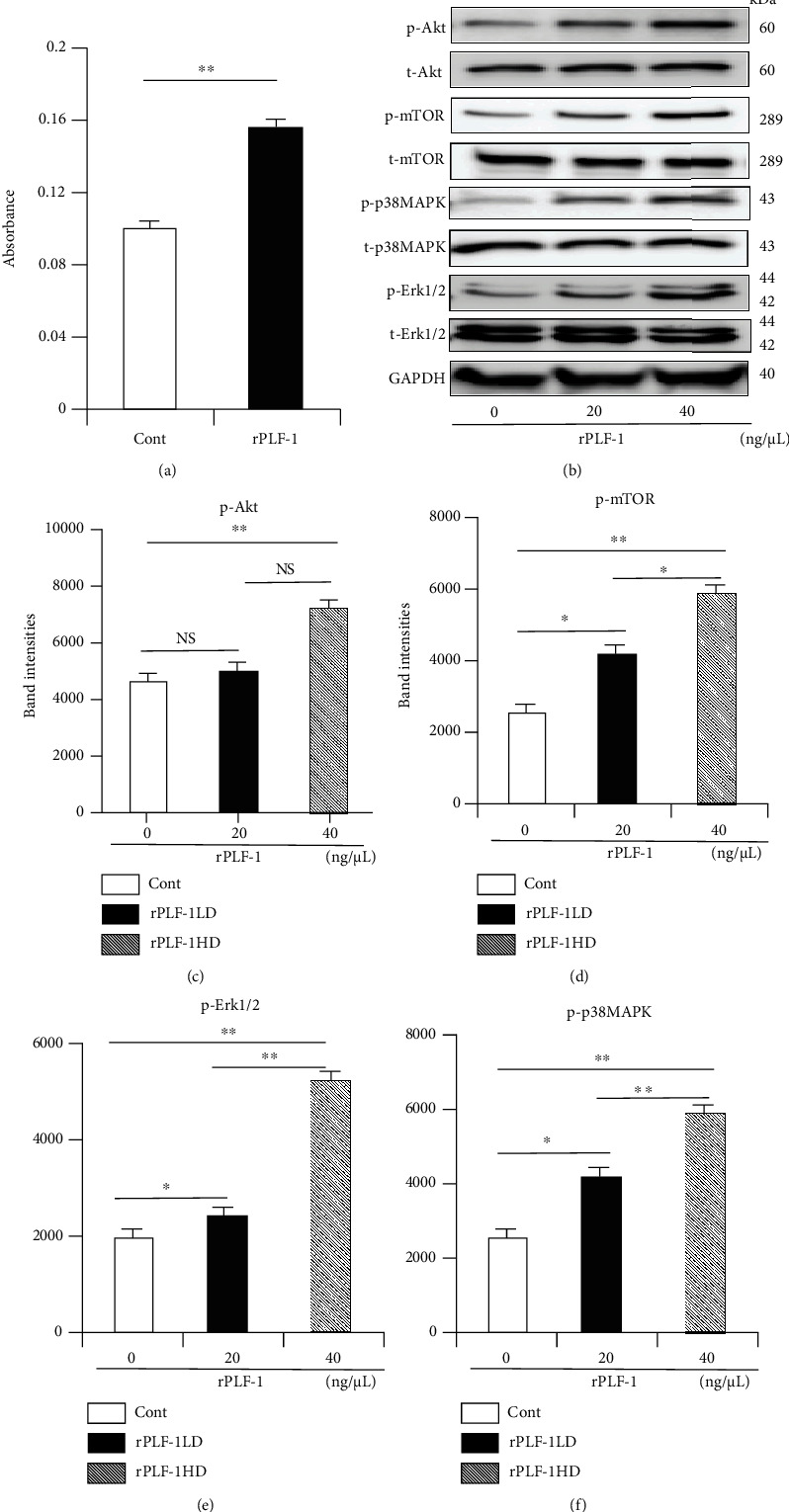
rPLF-1 promotes muscle cell proliferation. (a) C2C12 myoblasts were treated with rPLF-1 (100 nM) for 24 hr for proliferation. (b) Representative immunoblots and quantitative data show the dose-dependent levels of p-Akt, p-mTOR, p-p38MAPK, and p-Erk1/2 proteins in the cells. Results are mean ± SE (*n* = 3–6). ^∗^*p* < 0.05 and ^∗∗^*p* < 0.01 by Student's *t*-test or one-way ANOVA followed by Tukey's post hoc tests.

**Figure 13:**
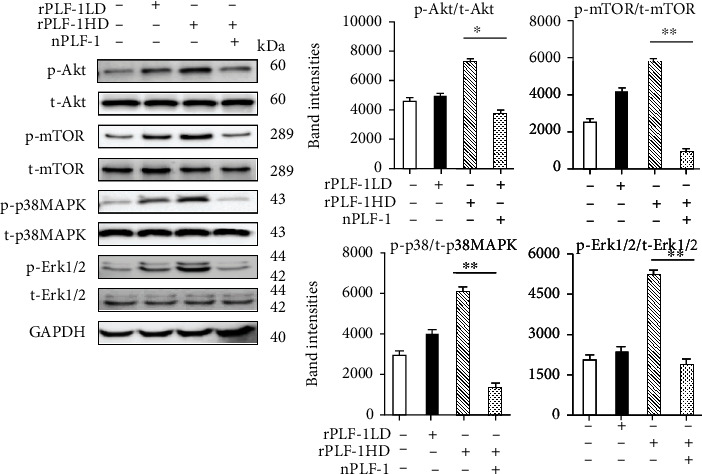
PLF-1 depletion suppressed cell growth signaling in C2C12 myoblasts in response to rPLF-1. The differentiated cells were cultured in the presence or absence of rPLF-1 and/or nPLF-1 for 45 min and then were subjected to a western blot assay. ^∗^*p* < 0.05 and ^∗∗^*p* < 0.01 by Student's *t*-test or one-way ANOVA followed by Tukey's post hoc tests.

**Figure 14:**
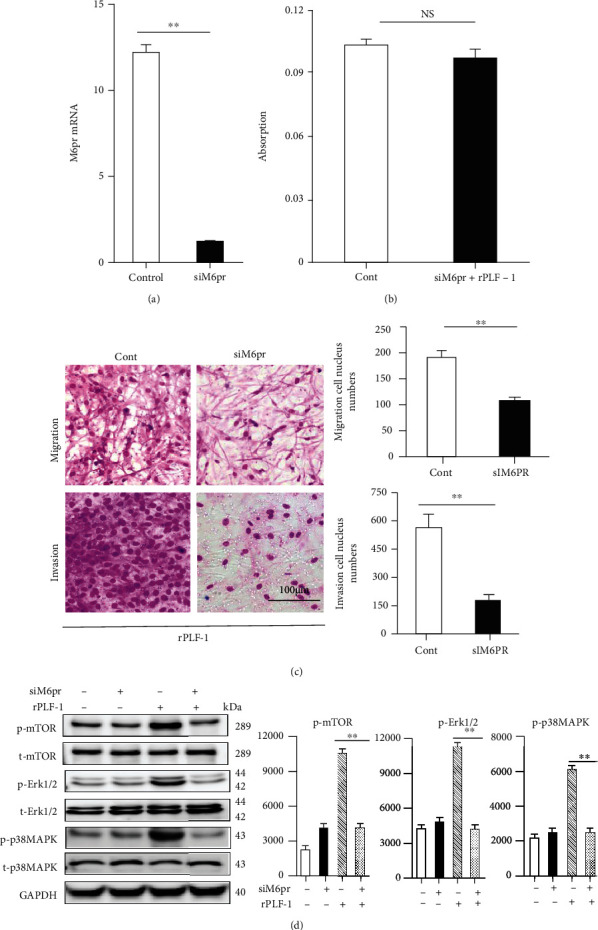
Silencing of M6pr impaired cellular functions and growth signaling in C2C12 myoblasts induced by rPLF-1. (a, b) Cells transfected with siM6pr for 48 hr were subjected to the analyses of cell proliferation, migration, and invasion with rPLF-1 (100 nM) (migration/invasion calculation: ×200 magnification). (c) Cells transfected with siM6pr for 48 hr were cultured in the presence or absence of rPLF-1 for 45 min and then were subjected to a western blot assay. Results are mean ± SE (*n* = 3–6). ^∗^*p* < 0.05 and ^∗∗^*p* < 0.01 by Student's *t*-test or one-way ANOVA followed by Tukey's post hoc tests.

To further explore the PLF-1/M6pr signaling pathway involved in muscle proliferation, we first sought to determine the proliferation ability of C2C12 myoblasts treated with rPLF-1.  Figure 12(a) shows the increased C2C12 myoblast proliferation ability in response to rPLF-1. Treatment with rPLF-1 was able to induce the phosphorylations of Akt, mTOR, p38MAPK, and Erk1/2 proteins in a dose-dependent manner (Figures 12(b)–12(f)). Interestingly, pretreatment with nPLF-1 completely diminished the rPLF-1-induced targeted growth signaling activation (Figure 13). Moreover, M6pr silencing impaired the rPLF-1-induced myoblast proliferation as well as the cell migration and invasion (Figures 14(a)–14(c)). Similar to nPLF-1, siM6pr blocked the rPLF-1-induced phosphorylations of mTOR, p38MAPK, and Erk1/2 proteins (Figure 14(d)). Thus, a PLF-1/M6pr axis appears to modulate myoblast proliferation via the activation of Akt/mTOR and p38MAPK/Erk1/2 signaling.

## 4. Discussion

This study contributes to the novel finding that the PLF-1 gene responds to a necrotic injury caused by CTX injection. We observed that rPLF-1 supplementation ameliorated the CTX-induced skeletal muscle myofiber loss, fibrosis, and proliferation associated with the activation of growth signaling (p-Akt/mTOR and p-p38MAPK/p-Erk1/2), leading to an improvement of muscle dysfunction in mice. PLF-1 also stimulated the production and mobilization of bone marrow MuSCs and muscle regeneration, contributing to the muscle morphological and functional improvements. Conversely, PLF-1 depletion with nPLF-1 delayed the CTX-related muscle damage and dysfunction. In C2C12 cells, the depletion of PLF-1 or the silencing of M6pr, respectively, decreased downstream proliferation-related signaling molecules (p-Akt, p-mTOR, p-GSK3*α*/*β*, p-Erk1/2, and p-p38MAPK) and cellular functions (migration/invasion and proliferation), providing evidence of a mechanistic explanation of the PLF-1/M6pr-mediated modulation of the skeletal muscle loss and mitigation of injury-associated muscle remodeling. To the best of our knowledge, this is the first study to provide evidence that PLF treatment may induce skeletal muscle regeneration under our experimental conditions.

Signal transducer and activator of transcription 5A (STAT5A) has been shown to bind to the PLF-1 promoter region [31]. The autocrine activity of PLF-1 can regulate angiogenesis by STAT5A transcriptional factor activation in mice [31]. In endothelial cells, the silencing of PLF-1 expression by short hairpin RNA or the depletion of PLF-1 activity with neutralizing antibodies results in a loss of the STAT5A-dependent proangiogenic activity of the conditioned medium [31]. Our present experiments demonstrated that PLF-1 gene expression in the mouse gastrocnemius muscle was increased in response to CTX. Conversely, PLF-1 blocking delayed the skeletal muscle regeneration, impaired the performance of the mice, and decreased the growth signal activation (p-Akt, p-mTOR, p-GSK3*α*/*β*, p-Erk1/2, and p-p38MAPK). We observed that nPLF-1 accelerated the CTX-induced muscle mass loss, interstitial fibrosis, and muscle dysfunction and impaired the growth signaling activation in mice. In agreement with these in vivo observations, our in vitro experiments showed that rPLF-1 increased the cell proliferation ability of C2C12 myoblasts, in association with the induction of the dose-dependent phosphorylations of Akt, mTOR, p38MAPK, and Erk1/2 proteins; all these effects were diminished by nPLF-1. Interestingly, M6pr silencing impaired the rPLF-1-induced cell proliferation as well as the cell migration and invasion. Moreover, similar to nPLF-1, siM6pr suppressed the rPLF-1-induced phosphorylations of the mTOR, p38MAPK, and Erk1/2 proteins. These results thus provide evidence that a PLF-1/M6pr axis acts as a key mediator of the muscle protective actions against CTX injury.

In inflammatory and metabolic cardiovascular and muscle diseases with injuries, the engagement of Toll-like receptors on the cell plasma membrane by their specific ligands leads to enhanced levels of inflammatory cytokines and chemokines (e.g., TNF-*α*, MCP-1, and IL-1*β*) [16, 37–39]. The ability of PLF-1 to decrease plasma TNF-*α* and IL-1*β*levels is like to contribute to skeletal muscle mass loss and fibrosis under our experimental conditions. We have shown herein that rPLF-1 supplementation will promote muscle regeneration and dysfunction in CTX-injured mice. Our findings revealed that rPLF-1 significantly decreased the levels of plasma inflammatory cytokines (IL-1*β* and TNF-*α*) and elevated the level of an important anti-inflammatory cytokine (IL-10) in response to CTX injection on day 3. In contrast, nPLF-1 produced an elevation in the levels of plasma IL-1*β*/TNF-*α* and IL-10 and resulted in smaller muscle fibers and extensive fibrosis and muscle dysfunction induced by CTX. Toll-like receptor 2 has been shown to modulate the expression of PLF-1 in vascular smooth muscle cells via the cathepsin K-mediated caspase-8 activation pathway both in vivo and in vitro [32]. We also demonstrated that PLF-1 overexpression with its plasmid promoted neointimal hyperplasia in response to a ligation injury and in response to a combination of ligation and cuff replacement injury; these effects were diminished by PLF-1 blocking [32]. We thus proposed that PLF-1 functions as an important mediator of injury-related skeletal muscle regeneration with inflammatory actions.

The function and the numbers of bone marrow-derived MuSCs are modified by various pathophysiological conditions, such as injury- and aging-related muscle diseases, and by therapeutic exercise interventions [14, 19]. In the present study, treatment with rPLF-1 resulted in an increase in the numbers of bone marrow and blood CD34^+^/integrin-*α*7^+^ MuSCs. The muscle tissues of the rPLF-1 mice exhibited strong desmin protein expression and organized laminin. The rPLF-1 treatment also elevated the numbers of integrin-*α*7^+^/CD34^+^ MuSCs in the mouse gastrocnemius muscles. In addition, the rPLF-1-treated mice had increased numbers of Ki67^+^/integrin-*α*7^+^ proliferating stem cells in injured muscles compared to control muscles. Conversely, nPLF-1 treatment had harmful effects on bone marrow MuSC production and mobilization and homing into injured muscles. Thus, the ability of PLF-1 to promote MuSC production and mobilization has a salutary effect on skeletal muscles under injury-related conditions by reducing inflammation, thereby enhancing muscle regeneration.

It should be noted that skeletal muscle-resident stem cells (also known as muscle satellite cells) contribute to muscle regeneration in response to various injuries [40–47]. However, a previous study demonstrated that lifelong reduction of satellite cells neither accelerated nor exacerbated sarcopenia and that satellite cells did not contribute to the maintenance of muscle size or fiber type composition during aging, but that their loss may contribute to age-related muscle fibrosis [48]. On the other hand, recent accumulating evidence indicates that bone marrow- (BM-) derived cells participate in skeletal muscle regeneration in several animal models [13–15]. In recent experiments, we found that transplantation of BM cells from GFP^+^ green mice to senescence-accelerated mouse prone 10 (SAMP10) mice provided direct evidence that BM-derived MuSCs also contribute to aged muscle regeneration (unpublished data), suggesting that the beneficial muscle effects of rPLF-1 are likely attributable, at least in part, to amelioration of BM-derived muscle stem cell regeneration capacity and muscle dysfunction in our experimental model. This concept is further supported by the previous findings of our group that BM-derived CD34^+^/*α*7^+^ MuSCs exhibited long-term effectiveness on the regeneration of aged muscle and reversal of aged muscle dysfunction in SAMP10 mice [3]. Interestingly, recent studies have picked up an interaction between BM-derived stem cells and muscle-resident satellite cells in skeletal muscle regeneration. Cerquone Perpetuini and colleagues [49] reported that Group I Pak inhibitor IPA-3 impaired myogenin expression and myotube formation in vessel-associated myogenic progenitors, C2C12 myoblasts, and satellite cells. The authors also observed that IPA-3 reduces p38*α*/*β* phosphorylation that is required to proceed through various stages of satellite cell differentiation: activation, asymmetric division, and ultimately myotube formation. It has been reported induction of bone marrow-derived cells' miogenic identidy by their interactions with satellite cell nitch (50) [50]. Further studies will be needed to explore the close interaction between BM-derived stem cells and muscle-resident satellite cells during the skeletal muscle regeneration process.

One of the practical implications of our present findings is that rPLF-1 administration appears to be a novel and attractive approach for preventing injury-related muscle disease. Our results clearly revealed the potential efficacy of rPLF-1 in the management of muscle mass loss and fibrosis and muscle dysfunction after injury. Based on our observations of a PLF-1 blocking-mediated delay in muscle regeneration, we propose that PLF-1 might be a novel molecular therapeutic target for skeletal muscle tissue wound healing and regeneration. Another implication of this study is that increased blood PLF-1 has potential as a biomarker to predict muscle injury in mice under our experimental conditions.

Some study limitations should be addressed. The MRP/PLFs are a family of highly homologous growth factor-inducible secondary response genes [23]. Unfortunately, many approaches, including the CRISPR-Cas9 technology used by our group and other research groups, have failed to generate total body or muscle-specific knockout mice [32], and although we used rPLF-1/nPLF-1 and siM6pr in the present investigation to explore the role of a PLF-1/M6pr axis in muscle remodeling and dysfunction in vivo and in vitro, we cannot fully define the exact role of PLF-1/M6pr in either skeletal muscle apoptosis or BM-derived MuSC mobilization, differentiation, and regeneration during disease-associated injury.

In summary, this newly discovered PLF-1-mediated modulation of the skeletal muscle mass and the amelioration of muscle regeneration has profound implications for our understanding of skeletal muscle biology and dysfunction management in response to injury in humans and animals. Our findings indicate that PLF-1 may have potential utility in the treatment or control of muscle loss and dysfunction in injury-related muscle disorders.

## Data Availability

All data used to support the findings of this study are included within the article. All data used to support the findings of this study are available from the corresponding author upon request.

## Abbreviations

ANOVA: Analysis of variance
CHO: Chinese hamster ovary
CTX: Cardiotoxin
ELISA: Enzyme-linked immunosorbent assay
Erk1/2: Extracellular signal-regulated kinase 1/2
FITC: Fluorescein isothiocyanate
GAPDH: Glyceraldehyde 3-phosphate dehydrogenase
GSK3*α*/*β*: Glycogen synthase kinase 3*α*/*β*
IL-1*β*: Interleukin-1*β*
M6pr: Mannose-6-phosphate receptor
mTOR: Mammalian target of rapamycin
MuSC: Skeletal muscle stem cell
nPLF-1: Neutralizing antibody against proliferin-1
OTC: Optimal cutting temperature
PCNA: Proliferating cell nuclear antigen
PCR: Polymerase chain reaction
p38MAPK: p38 mitogen-activated protein kinase
PLF-1: Proliferin-1
rPLF-1: Recombinant proliferin-1
STAT: Signal transducer and activator of transcription
TNF-*α*: Tumor necrosis factor-*α*.
